# 2-[^18^F]FDG PET in the Management of Radioiodine Refractory Differentiated Thyroid Cancer in the Era of Thyrosin-Kinases Inhibitors: A Real-Life Retrospective Study

**DOI:** 10.3390/diagnostics12020506

**Published:** 2022-02-16

**Authors:** Stefano Gay, Stefano Raffa, Anna De’Luca di Pietralata, Matteo Bauckneht, Lara Vera, Alberto Miceli, Manuela Albertelli, Silvia Morbelli, Massimo Giusti, Diego Ferone

**Affiliations:** 1 Endocrinology Unit, IRCCS Ospedale Policlinico San Martino, 16132 Genoa, Italy; stefano.gay89@gmail.com (S.G.); lara.vera@hsanmartino.it (L.V.); manuela.albertelli@unige.it (M.A.); 2 Endocrinology Unit, Department of Internal Medicine and Medical Specialties (DIMI), University of Genoa, 16132 Genoa, Italy; anna20deluca@gmail.com (A.D.d.P.); magius@unige.it (M.G.); 3 Nuclear Medicine Unit, IRCCS Ospedale Policlinico San Martino, 16132 Genoa, Italy; stefanoraffa@live.com (S.R.); matteo.bauckneht@gmail.com (M.B.); albertomiceli23@gmail.com (A.M.); silviadaniela.morbelli@hsanmartino.it (S.M.); 4 Department of Health Sciences (DISSAL), University of Genoa, 16132 Genoa, Italy

**Keywords:** 2-[^18^F]FDGPET/CT, radioiodine refractory thyroid cancer, prognosis

## Abstract

Purpose To evaluate the role of 2-[^18^F]FDGPET/CT in the follow-up of radioiodine refractory thyroid cancer (RR-TC). Methods Forty-six 2-[^18^F]FDGPET/CT scans from 14 RR-TC patients were considered. Thyroid function tests: thyroglobulin (Tg), levothyroxine (LT4), and tyrosine-kinases inhibitors (TKIs) assumptions were recorded. Metabolic tumour volume (MTV) and total lesion glycolysis (TLG) were calculated from each scan and correlated with clinical parameters and the overall survival (OS). Results Baseline TLG and MTV predicted OS (*p* = 0.027 and *p* = 0.035), and negative correlation with OS was also confirmed when the same parameters were measured in follow-up scans (*p* = 0.015 and *p* = 0.021). Tg also correlated with the OS; (*p* = 0.014; *p* = 0.019 and *p* = 0.009). However, TLG and MTV were not significantly correlated with Tg levels. MTV and TLG variation in time were reduced during TKI therapy (*p* = 0.045 and *p* = 0.013). Conclusions 2-[^18^F]FDGPET/CT confirmed its prognostic role at the first assessment and during the follow-up of RR-TC patients. 2-[^18^F]FDGPET/CT parameters seem at least partially independent from Tg. TKI therapy resulted in a measurable effect on the variation of 2-[^18^F]FDGPET/CT parameters over time.

## 1. Introduction

Advanced or metastatic radioiodine-refractory thyroid cancer (RR-TC) is a rare entity and its definition is still evolving [1]. To date, RR-TC is defined as a follicular-cell derived thyroid cancer no longer able to trap radioiodine or showing preserved radioiodine avidity only in some sites, or even displaying progression despite ^131^I treatments [1,2]. Its clinical management is therefore challenging, resulting in a poor prognosis [2,3,4,5]. The availability of tyrosine-kinases inhibitors (TKIs), in particular lenvatinib and sorafenib, radically changed the therapeutic approach to RR-TC, achieving in many cases the reduction of tumour burden and significantly improving the progression free survival (PFS) [6,7,8]. On the other hand, the efficacy of TKIs has to be balanced with their side effects, which could lead to dose reductions or even temporary or permanent drug discontinuation in a significant number of patients [9,10].

On this basis, effective follow-up strategies and imaging techniques are needed in order to assess progression, response rate, and duration, and to better define patients’ management. Computed tomography (CT) scan represents the gold standard imaging technique both at the time of therapy initiation and during the follow-up [2,3,4,5], and tumour shrinkage assessed through RECIST 1.1 criteria is considered to measure the response to TKIs [11].

On the other hand, thyroglobulin (Tg) concentration and doubling time (Tg-DT) proved to have a prognostic value in RR-TC [12,13], and it is presently used as a complementary tool in monitoring drug response profile. Nevertheless, in some cases, Tg could lose its reliability as a marker of disease, due to the possibility of cell dedifferentiation.

Of note, Tg variations are not always consistent with CT scans findings. In particular, after TKIs initiation, the drop of the Tg is not always consistent with the radiological response, and conversely a CT documented progressive disease might also not be associated with the raising Tg levels [14].

In this scenario, [^18^F]-fluorodeoxyglucose positron emission tomography (2-[^18^F]FDGPET)/CT could improve prognostic stratification of patients with RR-DTC, providing insights into tumour glucose consumption and aggressiveness [15]. In the clinical practice, the report of 2-[^18^F]FDGPET is most often based on a visual/qualitative assessment and further supported by the use of SUVmax as a readily available semi-quantitative measure [16]. However, other 2-[^18^F]FDGPET-derived parameters have been proposed to better capture the extent (metabolic volume of the tumour, MTV) and intensity (total lesion glycolysis, TLG) of the metabolically active disease burden. These variables have shown a potential additional value in several oncological diseases, even in those which are not characterized by high metabolic activity at disease onset, including DTCs [15]. Ahmaddy et al. even suggested that 2-[^18^F]FDG -PET/CT might outweigh the CT scan in the evaluation of the treatment response in patients with advanced RR-DTC undergoing TKI therapy. These authors claimed 2-[^18^F]FDGPET may play a role in the early assessment of the response to the treatment, identifying those patients who will most likely benefit from it [17]. Similar findings were reported by Valerio et al., showing basal 2-[^18^F]FDGPET to be predictive of the response to TKIs and correlated with the OS [18].

Given these premises, we aimed to retrospectively evaluate the predictive value of a serial assessment of 2-[^18^F]FDGPET/CT parameters in a cohort of RR-TC patients, and their correlation with Tg and thyroid function tests.

## 2. Materials and Methods

All 2-[^18^F]FDGPET/CT performed in RR-TC patients between 2009 and 2019 in IRCCS Policlinico San Martino Hospital were evaluated. At the time of each examination, thyroid axis as well as Tg values were recorded, and the ongoing therapies were reported, with specific regard to the levothyroxine (LT4) dosage and TKIs administration. Patients’ clinical history and previous cancer treatments were also recorded, and data concerning the overall survival after the exam were subsequently registered.

All the patients signed a written informed consent before each examination. Data collection, as well as the subsequent analysis, were performed in compliance with the 1964 Helsinki Declaration. Regional Independent Ethical Committee (IRB) approved the study.

### 2.1. 2-[^18^F]FDGPET/CT Acquisition

2-[^18^F]FDG PET/CT was performed according to the international guidelines (16) using a 16-slices PET/CT hybrid system (Biograph 16, Siemens Medical Solutions, Knoxville, TN, USA). Briefly, patients fasted overnight prior to the intravenous administration of 300–400 MBq of FDG, which was performed in a quiet room, with the patient lying in a recumbent position and instructed not to move. Blood glucose was measured before tracer injection, as to ensure blood glucose levels <160 mg/dL. To minimize artifacts caused by the urinary tract, patients were asked to drink 500 mL of water 1 h prior to image acquisition and to empty the bladder just before the acquisition start. Imaging started 60 ± 15 min after intravenous tracer administration. The technical parameters of the 16-detector row, helical CT scanner included a gantry rotation speed of 0.5 s and table speed of 24 mm per gantry rotation. The PET component of the combined imaging system had an axial view of 16.2 cm per bed position, with an interslice spacing of 3.75 mm. The trans-axial field of view and pixel size of the reconstructed PET images were 58.5 cm and 4.57 mm, respectively, with a matrix size of 128 × 128. Unenhanced low-dose CT was performed at 140 kV and 40 mA for attenuation correction of emissive data and anatomical localization of PET dataset. An emissive scan was performed in 3D mode, shortly after CT acquisition, with a 3-min acquisition per bed position. PET sinograms were reconstructed by means of ordered-subset expectation maximization (OSEM) iterative reconstruction algorithm (three iterations, eight subsets). A scan was performed starting from the orbital plane on to the mid-thigh, except for the cases where the clinical history demanded a whole body, vertex-to-toes scan.

### 2.2. Image Analysis

2-[^18^F]FDGPET/CT images were interpreted in consensus by two expert nuclear medicine physicians blinded to biochemical and clinical results, as well as to the results of other imaging procedures. From the attenuation-corrected FDG PET images, the maximum standardized uptake value (SUVmax) of the hottest lesion was obtained in the transaxial view. Further, a volume of interest was drawn using an SUV-based automated contouring program (Syngo Siemens workstation, Siemens Medical Solutions, Princeton, NJ, USA) with an volumetric region of interest based on a 3D isocontour at 41% of the maximum pixel value (SUVmax), as previously recommended [16]. Total Metabolic Tumor Volume (MTV) was obtained by the sum of MTV values of all patients’ lesions. Total Lesion Glycolysis (TLG) was computed as the sum of TLG of every lesion for each patient (thus corresponding for each patient, to the sum of the VOI average/mean SUV value for each lesion multiplied by corresponding MTV).

### 2.3. Laboratory Tests

Serum Tg was assayed through immuno-chemiluminescence (Roche Diagnostics, Mannheim, Germany). Analytical sensitivity of the method was 0.04 ng/mL. TSH and fT4 were measured by means of ultrasensitive immuno-chemiluminescence methods (Roche Diagnostics). Normality ranges were 0.3–4.2 mIU/L for TSH and 9.3–17.0 pg/mL for fT4.

### 2.4. Statistical Analysis

Statistical analysis was carried out by means of MedCalc Portable Launcher software, version 2.2.0.0; the same program was used to create all figures and graphs. Parametric distribution of the variables was assessed through the Kolmogorov–Smirnov test, and data were reported as mean ± standard deviation (95% CI of the mean) if parametric, or median (range, 95% CI of the median) if non-parametric.

The association among non-quantitative data were assessed through the Chi-squared test. Cox proportional hazard regression analysis was used to evaluate the association between each variable and the OS, and Kaplan–Meier curves to assess the difference of OS among groups. The correlation among quantitative variables was assessed by means of Spearman rank correlation test, while linear regression test was used to investigate the relationship between thyroid function tests, LT4, Tg, and 2-[^18^F]FDGPET/CT parameters. As regards the TSH, it was considered as a continuous rather than a dicotomic variable (suppressed/unsuppressed); for this reason, patients with unsuppressed TSH were included in the analysis together with those who had it suppressed. In order to assess 2-[^18^F]FDGPET/CT parameters variation, the ratio between the values registered at the subsequent and the previous scan was considered; this parameter could not be assessed for patients who had a single 2-[^18^F]FDGPET/CT scan. Mann–Whitney test was used to compare 2-[^18^F]FDGPET/CT parameters variation in the periods in which TKI was administered to those in which no therapy was given. Lastly, sensitivity and specificity in predicting 1-year OS were calculated by means of ROC curves. *p* values < 0.05 were considered as statistically significant.

## 3. Results

Overall, out of 684 patients followed between 2009 and 2019, 46 2-[^18^F]FDGPET/CT scans were collected from 14 patients. Out of them, five were females, the mean age at diagnosis was 65.0 (±12.2, 95% CI 57.9 to 72.0), while at the time of the first scan it was 69.7 (±10.6, 95% CI 63.5 to 75.8). Surgery was performed in all but one patient: a total thyroidectomy in the majority of cases (64.3%), combined with central neck compartment lymphadenectomy in 7.1% or central and lateral neck compartment lymphadenectomy in 21.4%. In one elderly patient, surgery was not performed due to the local extension of the disease and the clinical status; therefore, only external radiotherapy was delivered.

The most prevalent histology was follicular thyroid cancer (eight patients), while a papillary carcinoma was reported in four patients, and poorly differentiated thyroid cancer and the combination of papillary and Hurtle cell thyroid cancer in one patient each.

Radioiodine therapy was performed in all surgically-treated patients, with an average cumulative dose of 386.4 mCi (±292.4, 95% CI 200.6 to 572.2 mCi).

### 3.1. First 2-[^18^F]FDGPET Scan Assessment

At the time of the first scan, the median Tg was 1519.5 ng/mL (0.04 to 25,454.0, 95% CI 23.9 to 5788.6 ng/mL) and the mean administered LT4 dosage was 903.6 mcg/week (±298.7, 95% CI 731.1 to 1076.0 mcg/week). All but three subjects had suppressed TSH levels (median 0.093 mU/L, 0.005 to 4.700 mU/L, 95% CI 0.014 to 1.047 mU/L), and the mean fT4 was 17.46 pg/mL (±3.99, 95% CI 15.16 to 19.77 pg/mL).

The first 2-[^18^F]FDGPET was performed after a median of 29 months (range 3–416 months, 95% CI 5.13–104.37 months) from the diagnosis and LT4 therapy initiation. The distribution of the 2-[^18^F]FDGPET/CT exams among the study population is described in Table 1. As regards the initial assessment, median MTV at the first 2-[^18^F]FDGPET/CT scan was 18.90 cm^3^ (range 0.71–1197.3 cm^3^; 95% CI 3.52 to 58.87 cm^3^), while median TLG was 94.74 (5.33–10,632.20; 95% CI 23.71 to 644.75 SUV mean × cm^3^).

### 3.2. TKI Treatment

Lenvatinib was administered to 11 patients during the study period; among them, 8 had had a previous line of systemic therapy with sorafenib.

At the time of the data collection, 11 patients had died due to thyroid cancer progression, 2 were still in treatment with lenvatinib, and 1 had withdrawn lenvatinib therapy due to adverse events. Overall, three patients received sorafenib as the only systemic therapy, three had only lenvatinib, and eight received both the drugs.

A representation of TKI administration schedule with respect to the study period is provided in Table 2.

### 3.3. Overall Survival

The median OS from the first 2-[^18^F]FDGPET/CT scan was 25 months (range 7–53, CI 95% 12.92 to 39.35 months), while the follow up of the patients who were still alive at the time of data collection was 45, 31, and 44 months, respectively.

No association was found between the OS and histology (*p* = 0.349), type of surgery (*p* = 0.586), and administered radioiodine dosage (*p* = 0.545).

Conversely, cox proportional hazards regression analysis showed a predictive role of TLG (*p* = 0.027) and MTV (*p* = 0.035), performed at baseline, on the OS, while Tg values nearly approached statistical significance (*p* = 0.083). On the other hand, a negative correlation was found between basal Tg values at the first analysis and the OS (*p* = 0.036).

Differently from Tg (*p* = 0.145), TLG and MTV proved predictive of 1-year mortality (sensitivity 60%, specificity 100%, criterion > 817.8, AUC 0.812, *p* = 0.001; sensitivity 60%, specificity 96%, criterion > 126.7 cm^3^, *p* = 0.001, AUC 0.815).

The results of Cox regression analysis are listed in Table 3, whereas the sensitivity and specificity of each parameter in predicting 1-year-mortality are reported in Table 4.

**Figure 1:**
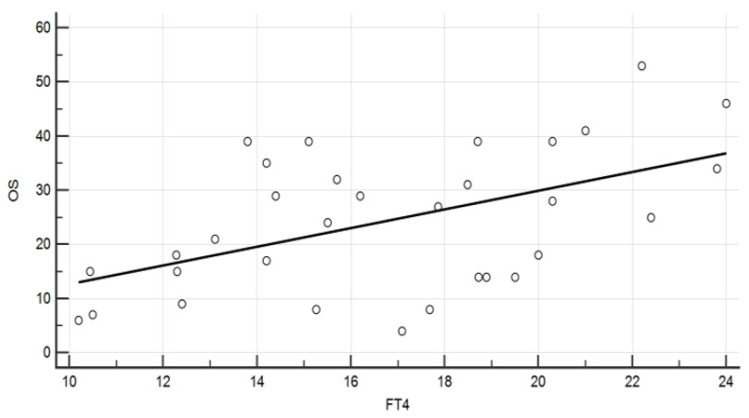
Diagram showing the association between fT4 levels (pg/mL) and the overall survival (months) (Spearman rank correlation, ρ = 0.457, *p* = 0.009).

Data from the whole 46 evaluations showed a negative correlation of both TSH and Tg levels with the OS (*p* = 0.014 and *p* = 0.019.) A positive correlation was recorded, instead, between the OS and fT4 (*p* = 0.009, Figure 1).

**Figure 2:**
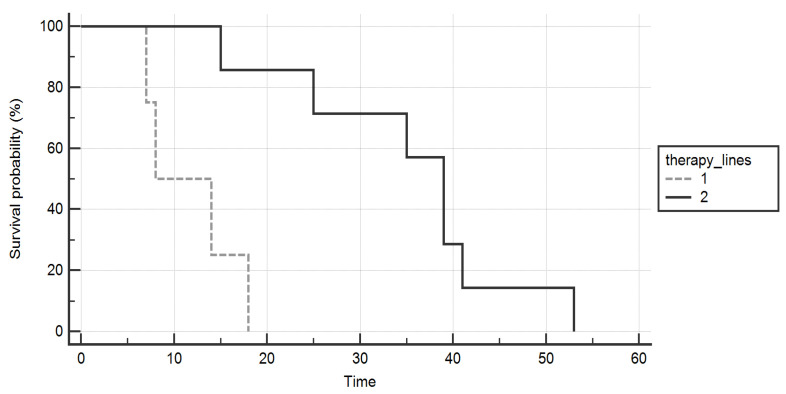
Kaplan–Meier curves comparing the overall survival (months) of patients who were treated with two lines of tyrosine-kinases inhibitors (continuous line) versus those who received only one (dashed line). A significant gain was proved in the formers.

An improved overall survival was found in the group of patients who underwent two lines of TKIs compared to those who received only one (*p* = 0.005, Figure 2).

### 3.4. 2-[^18^F]FDGPET/CT Parameters Correlations

As regards 2-[^18^F]FDGPET/CT parameters, both TLG and MTV proved a negative correlation with the OS (*p* = 0.015 and *p* = 0.021, respectively).

Linear regression analysis showed an association between MTV values and TSH (R^2^ = 0.19, *p* = 0.006) and between MTV and fT4 (R^2^ = 0.13, *p* = 0.029). Conversely, Tg was not related to MTV (*p* = 0.869).

Also, TLG was associated to TSH and fT4 (R^2^ = 0.22, *p* = 0.005 and R^2^ = 0.12, *p* = 0.044, respectively).

No correlation was found between Tg and both MTV and TLG (*p* = 0.807 and *p* = 0.467, respectively). However, a correlation was found between Tg and MTV variation in time (*p* = 0.011), while not between Tg and TLG variation (*p* = 0.118).

**Figure 3:**
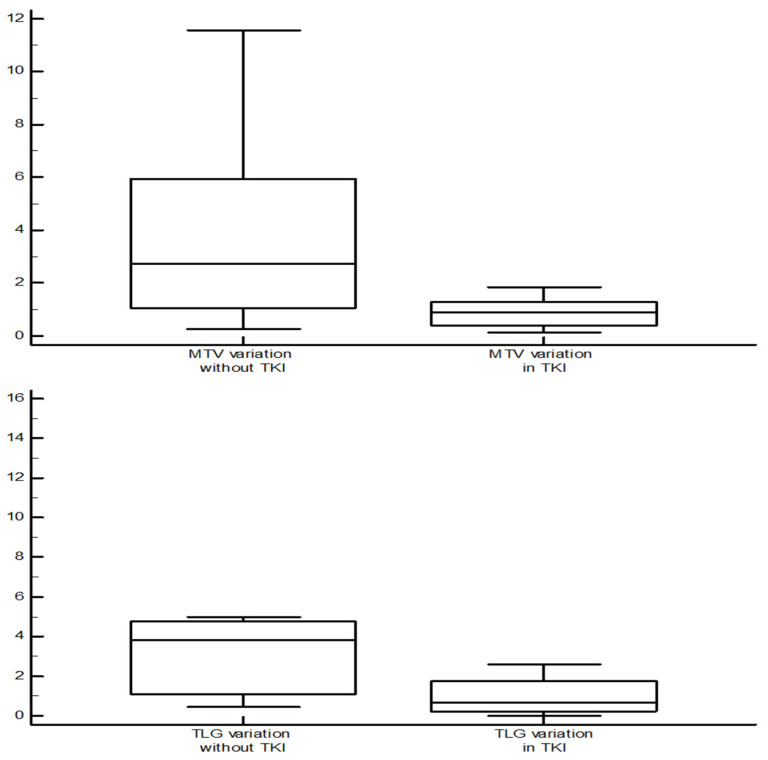
Comparison of MTV and TLG variation (value at the subsequent evaluation/value at the previous evaluation) between the scan performed during tyrosine-kinases inhibitors therapy and those without systemic therapy. In the first group, both MTV and TLG showed a trend of reduction, while in the second one it tended to increase (Mann–Whitney test, median 0.88 vs. 2.73 cm^3^, *p* = 0.045, for MTV; median 0.65 vs. 4.01, *p* = 0.013 for TLG).

A difference in terms of MTV variation was found between patients in treatment with TKIs and those who were not (median 0.88 vs. 2.73 cm^3^, *p* = 0.045). Similar findings were recorded for TLG (median 0.65 vs. 4.01, *p* = 0.013) and Tg variations (median 1.24 vs. 2.95, *p* = 0.047) (Figure 3).

## 4. Discussion

In our retrospective analysis, we evaluated the role of 2-[^18^F]FDGPET/CT in the management of RR-TC patients, in addition to Tg and CT scan assessment, which represent the gold standard tools for the follow-up of these patients [2,4]. Interestingly, 2-[^18^F]FDGPET/CT seems to provide information about the clinical course of the disease through a first single scan. This may be due to the insights about the cellular biological activity besides the anatomical tumour burden [19]. In this perspective, similar findings were obtained by Manohar et al. in their analysis, which included 62 RR-TC patients: MTV and TLG proved related to both the PFS and the OS, as well as Tg and Tg-DT [15].

On the other hand, differently from the abovementioned study, the present one was also aimed at investigating the performance of 2-[^18^F]FDGPET/CT during the follow-up, and even in this other setting the association with the OS was maintained.

A further intent of our analysis was to assess the impact on TKIs therapy on 2-[^18^F]FDGPET/CT parameters. In this regard, our data suggest a completely opposite trend in the evolution of the 2-[^18^F]FDGPET/CT parameters when patients were treated with TKIs compared to the periods in which the same patients were not. Particularly, for both MTV and TLG, a trend in reduction seemed to be present in the former (median of the ratios 0.88 and 0.65, respectively), while an increase was recorded in the latter (2.73 and 4.01, respectively), confirming the effect of these drugs in hindering cancer cell biology.

Data recently published by Valerio et al. seems consistent with these findings, showing a metabolic response in most of the patients who started lenvatinib and an improved OS in this group compared to those who did not achieve a response. In that study, on the other hand, a concomitant shrinkage was registered in the tumoral lesions of 22 out of the 24 patients showing a metabolic response, and in 60.6% of patients this was correlated with the biochemical response [18].

Data concerning cancer cell activity and metabolism, indeed, may represent an important tool especially when biochemical and morphological data diverge [14,20,21]. In most patients, a drop in Tg levels is observed after TKI initiation, but it is not always followed by a tumour size reduction at the CT scan [14,22]. On these bases, a multiparametric approach, comprehensive of either morphological, biochemical or metabolic data, could be advisable in evaluating tumour evolution in time. Moreover, while PET-determined non-responders proved lower PFS and disease-specific survival, only this last parameter resulted in association with the therapeutic failure according to RECIST [17].

Another aspect worth mentioning and which is still a matter of discussion, is the suitability of a further line of TKI after the failure of the first one: Our findings seem to support the use of TKIs in second line, in line with the data reported by other authors on this topic [23,24].

A correlation between 2-[^18^F]FDGPET/CT parameters and Tg was mentioned by Chai et al. in 2017; they found higher stimulated Tg levels to be predictive of the optimal diagnostic accuracy of the exam [21]. Leboulleux et al. in 2009 reported similar findings and highlighted the role of recombinant TSH stimulation in improving diagnostic performance of 2-[^18^F]FDGPET/CT [25]. On the other hand, data from other studies did not support the correlation between TSH levels and PET finings in RR-TC. In their recent prospective analysis, Almeida LS et al. failed to find a significant difference in SUVmax and in diagnostic accuracy of 2-[^18^F]FDGPET/CT performed during TSH stimulation in hormonal withdrawal compared to those in TSH-suppression. However, in this case, the study population was very small, and the correlation between TSH and SUVmax almost reached the statistical significance (*p* = 0.064) [26]. Furthermore, the previous meta-analysis published in 2010 by Ma et al. stated the role of TSH stimulation in improving diagnostic performance of 2-[^18^F]FDGPET in patients treated for a RR-DTC, with elevated Tg levels and a negative whole-body scintigraphy (WBS) scan [27].

On the other hand, the current evidence in literature suggests that a chronic TSH suppression might delay disease recurrence and improve the OS [4,28], and the hereby highlighted relationship between thyroid function tests and 2-[^18^F]FDGPET/CT parameters seems in keeping with that. However, the numerosity of the present sample prevents any clear conclusion on this issue.

Our data failed, instead, in demonstrating an association between Tg levels and 2-[^18^F]FDGPET/CT parameters, while a correlation was proved between Tg and MTV variation in time. This could be an important point to support the complementarity of the information provided by these tests. Tg represents, indeed, a prognostic factor in these patients, and in particular a short Tg-DT is correlated with a reduced OS [12,29]. Furthermore, as previously mentioned, stimulated Tg levels should be considered in discriminating which patients may deserve an 2-[^18^F]FDGPET/CT or CT scan [21,29,30]. On the other hand, the loss of cellular differentiation in thyroid cancer may lead to the dissociation between Tg levels and tumour progression [31,32].

The small number of patients enrolled is the main limitation of the present study; this is due to the rarity of the disease, beside the fact that these patients do not regularly undergo 2-[^18^F]FDGPET/CT. Nevertheless, the availability of a strict and long-term follow-up, during which either clinical, biochemical, or radiological data are provided and recorded, strengthens the study. Interestingly, most male patients were included in the present study. In this framework, it should be noted that the female prevalence characterizing the well differentiated disease is not always maintained in the poor differentiated and/or more aggressive disease [33,34].

## 5. Conclusions

2-[^18^F]FDGPET/CT confirmed its prognostic role both in the initial assessment and during the follow-up of patients with RR-TC. MTV and TLG seem at least somehow independent from Tg values, while a relationship might be present with the thyroid axis parameters. TKIs confirmed their efficacy as systemic therapy even in second line. TKIs also seem to impact 2-[^18^F]FDGPET/CT parameters, limiting their evolution in time. Further studies might be carried out to investigate their effect even in the improvement of the OS. In this perspective, 2-[^18^F]FDGPET/CT should be considered an important tool during the follow-up, which could integrate the information provided by other morphological and biochemical tests.

## Figures and Tables

**Table 1:** Study population; clinical characteristics at baseline 2-[^18^F]FDGPET evaluation.

Patients	Age	Sex	Histology	TKI	TSH * (mU/L)	fT4 ** (pg/mL)	Tg ** (ng/mL)	LT4 (µg/Week)	MTV * (cm^3^)	TLG*	OS (Months)	N° FDG-PET
1	63	F	FTC	-	0.89	14.2	2283.0	1050	1.56	6.88	35	6
2	77	M	PTC	L	0.03	22.2	1.1	900	126.67	700.48	53	4
3	79	M	FTC	-	0.01	22.4	20,583.2	1100	31.13	377.92	25	2
4	67	M	FTC	S	0.15	18.7	26.3	1050	25.00	314.17	39	5
5	71	M	PTC	S	0.06	18.8	2237.1	1125	3.27	-	-	4
6	70	F	FTC	L	0.02	22.1	2014.5	850	0.71	5.34	-	1
7	43	M	FTC	S	0.01	21.0	698.9	1400	7.25	45.96	41	5
8	76	M	PTC	S	0.17	15.1	532.4	1050	3.55	27.16	39	5
9	81	M	HC+PTC	S	2.44	12.3	1025.2	225	50.98	-	15	2
10	68	F	FTC	S	0.13	19.5	8853.3	650	4.04	22.99	14	2
11	53	M	FTC	-	4.70	10.5	5431.7	1050	1197.32	10,632.20	7	3
12	77	F	FTC	S	0.01	17.7	25,454.2	825	238.88	1748.60	8	1
13	76	F	PDTC	-	2.41	12.3	0.1	450	27.44	143.51	18	2
14	75	M	PTC	-	0.01	17.8	3.8	925	12.80	44.42	-	4

(TKIs: tyrosine-kinases inhibitors; Tg: thyroglobulin; LT4: administered levothyroxine dosage per week; MTV: metabolic tumour volume; TLG: total lesion glycolysis; OS: overall survival); * approximated to two decimals; ** approximated to one decimal.

**Table 2:** The distribution of TKIs therapies among the study population and with regards to the study period.

	Before the Study Period	At the Time of the First ^18^F-FDG PET Scan	During the Study Period
1	sorafenib	no	lenvatinib
2	sorafenib	lenvatinib	lenvatinib
3	sorafenib	no	lenvatinib
4	sorafenib	sorafenib	lenvatinib
5	sorafenib	sorafenib	lenvatinib
6	lenvatinib	lenvatinib	-
7	sorafenib	sorafenib	lenvatinib
8	sorafenib	sorafenib	lenvatinib
9	sorafenib	sorafenib	lenvatinib
10	sorafenib	sorafenib	no
11	no	no	sorafenib
12	sorafenib	sorafenib	-
13	no	no	lenvatinib
14	no	no	lenvatinib

**Table 3:** Table reporting the results of Cox regression analysis of variables predicting the OS.

	Null Model-2 Log Likelihood	Full Model-2 Log Likelihood	Chi-Squared	DF	Significance Level
MTV	35.58	31.47	4.43	1	*p* = 0.035
TLG	30.78	25.92	4.86	1	*p* = 0.027
TSH	35.58	29.77	5.81	1	*p* = 0.016
fT4	35.58	31.59	3.99	1	*p* = 0.046
Tg	35.58	32.58	2.99	1	*p* = 0.083
RAI dosage	30.78	30.46	0.32	1	*p* = 0.545

**Table 4:** Table reporting the results of ROC curves analysis performed on all variables with respect to 1-year-survival. As regards the associated criterion, values are to be expressed in cm^3^ for the MTV, mU/L for the TSH, pg/mL for the fT4, ng/mL for the Tg, and mCi for the RAI dosage.

	AUC	Significance Level	Youden Index (J)	Associated Criterion	Sensitivity %	Specificity %
MTV	0.815	0.001	0.56	>126.67	60	96
TLG	0.812	0.001	0.60	>817.86	60	100
TSH	0.708	0.133	0.47	>0.47	67	81
fT4	0.763	0.010	0.54	≤17.68	100	54
Tg	0.675	0.145	0.48	>1253.0	75	73
RAI dosage	0.563	0.793	0.37	≤500	100	37

## Data Availability

The datasets generated during and/or analysed during the current study are available from the corresponding author on reasonable request.
